# Evaluation of skin dose and skin toxicity in patients undergoing intraoperative radiotherapy for early breast cancer

**DOI:** 10.1002/acm2.13338

**Published:** 2021-07-13

**Authors:** Jeannie Hsiu Ding Wong, Zainor Zaili, Rozita Abdul Malik, Anita Zarina Bustam, Marniza Saad, Suniza Jamaris, Joanne Aisha Mosiun, Nur Aishah Mohd Taib, Ngie Min Ung, Mee‐Hoong See

**Affiliations:** ^1^ Department of Biomedical Imaging Faculty of Medicine University of Malaya Kuala Lumpur Malaysia; ^2^ Clinical Oncology Unit Faculty of Medicine University of Malaya Kuala Lumpur Malaysia; ^3^ Breast Surgery Unit Department of Medicine, Faculty of Medicine University of Malaya Kuala Lumpur Malaysia; ^4^ Department of Surgery, Faculty of Medicine University of Malaya Kuala Lumpur Malaysia

**Keywords:** Dosimetry, Early breast cancer, INTRABEAM system, IORT, local complications, Radiochromic film, skin toxicity

## Abstract

**Purpose:**

This study aims to evaluate in vivo skin dose delivered by intraoperative radiotherapy (IORT) and determine the factors associated with an increased risk of radiation‐induced skin toxicity.

**Methodology:**

A total of 21 breast cancer patients who underwent breast‐conserving surgery and IORT, either as IORT alone or IORT boost plus external beam radiotherapy (EBRT), were recruited in this prospective study. EBT3 film was calibrated in water and used to measure skin dose during IORT at concentric circles of 5 mm and 40 mm away from the applicator. For patients who also had EBRT, the maximum skin dose was estimated using the radiotherapy treatment planning system. Mid‐term skin toxicities were evaluated at 3 and 6 months post‐IORT.

**Results:**

The average skin dose at 5 mm and 40 mm away from the applicator was 3.07 ± 0.82 Gy and 0.99 ± 0.28 Gy, respectively. Patients treated with IORT boost plus EBRT received an additional skin dose of 41.07 ± 1.57 Gy from the EBRT component. At 3 months post‐IORT, 86% of patients showed no evidence of skin toxicity. However, the number of patients suffering from skin toxicity increased from 15% to 38% at 6 months post‐IORT. We found no association between the IORT alone or with the IORT boost plus EBRT and skin toxicity. Older age was associated with increased risk of skin toxicities. A mathematical model was derived to predict skin dose.

**Conclusion:**

EBT3 film is a suitable dosimeter for in vivo skin dosimetry in IORT, providing patient‐specific skin doses. Both IORT alone and IORT boost techniques resulted in similar skin toxicity rates.

## 1 INTRODUCTION

For the past 30 years, multiple randomized control trials (RCTs) have shown that breast‐conserving therapy and mastectomy have similar recurrence and survival rates among breast cancer patients.^1^ Radiation therapy to the whole breast after breast‐conserving surgery (BCS) is indicated to reduce ipsilateral breast tumor recurrence (IBTR).^2^ A more recent and alternative approach is irradiating the tumor bed with a technique known as accelerated partial breast irradiation (APBI), which treats only the lumpectomy bed within a 1–2 cm margin.^3^, ^4^ Various APBI techniques have been developed, including multi‐catheter interstitial brachytherapy, balloon catheter brachytherapy, conformal external beam radiation therapy (EBRT), and intra‐operative radiation therapy (IORT).

Targeted IORT (TARGIT) using the INTRABEAM system delivers a single dose of 20 Gy using a 50 kV X‐ray to the tumor bed after excision. This technique showed promising results in phase two trial.^5^ In 2014, a multi‐center RCT showed that the use of IORT alone (TARGIT‐A) immediately after a lumpectomy within a risk‐adapted approach could achieve local control comparable to that of EBRT in patients with early breast cancer and low risk of recurrence.^6^

The advantages of IORT over whole breast irradiation or boost via EBRT include immediate radiotherapy during the surgery, direct irradiation of the tumor bed, reduced risk of geometrical miss, shorter treatment time, and reduced risk of radiation injury to the surrounding structure. Following the TARGIT‐A study, the TARGIT‐B studied IORT boost delivered as an alternative to EBRT boost dose.^7^

Radiation‐induced skin toxicity is dependent on the amount of radiation exposure. The onset of radiation‐induced skin toxicity varies from early to immediate and late treatment. Balter *et al*. (2010) defined the time of skin toxicity onset as prompt (<2 weeks), early (2–8 weeks), mid‐term (6–52 weeks), and long term (>40 weeks).^8^ The severity and onset of skin toxicity depend on the total dose received the interval between radiation exposure (dose fractionation vs. single large dose), and the irradiated area. Patients’ health, nutritional status, age, compromised skin integrity, and site of irradiated skin might also affect the expression of injury and recovery rate.

Notably, most of the previous IORT studies had studied the radiation exposure on tumor bed dose alone.^9^, ^10^, ^11^ At lower radiation exposure, prompt and early radiation effects may be observed, but these will heal. However, more severe radiation‐induced skin toxicities due to higher radiation exposure may have a later onset time and may take longer to heal.^8^ IORT delivers a high radiation dose (20 Gy) to the tumor bed in a single fraction. Thus, there is a need to investigate mid‐term skin toxicity. Only two studies have been conducted to investigate IORT acute and late skin toxicity, which showed minimal or no acute skin effects.^9^, ^11^ However, the skin dose was not directly evaluated in these studies. Hence, this paucity of information concerning skin dose has led to a poor understanding of the radiobiological effects. The skin dose must be directly measured during treatment so that an association (if any) between the dose received by the skin and skin toxicity may be assessed. In vivo measurement is the only method to assess the patient skin dose directly.

Several groups have attempted to measure in vivo skin dose using different radiation dosimeters, such as thermoluminescent dosimeters (TLD), radiochromic film, and optically stimulated luminescent dosimeter (OSLD).^12^, ^13^, ^14^, ^15^ However, due to variability in the type of dosimeter used and measured positions, a direct comparison between studies was difficult to evaluate. Radiochromic films such as Gafchromic EBT3 films are tissue equivalent and is the only dosimeter that can provide 2D dosimetric information.^16^, ^17^, ^18^, ^19^, ^20^ Films are very thin and tissue equivalent and are, therefore, more representative of skin dose.^14^ Lee *et al*. had reported that the breast volume, the ratio of applicator diameter and breast volume, and the distance between skin and tumor were significantly correlated with maximum skin dose.^12^ However, they used OSLD, which may have an energy dependence issue at 50 kV beam.^21^, ^22^ Hence, there is a scarcity of IORT studies with skin dose dosimetry and skin toxicity outcomes. Therefore, in this study, we calibrate the EBT3 film, determine the uncertainties of measurements, followed by in vivo skin dose evaluation of radiation doses during IORT. We also aim to determine the association of the skin dose with radiation‐induced skin toxicity.

## 2 MATERIALS AND METHODS

In this work, the Gafchromic EBT3 film (Ashland Inc., Wayne, NJ, USA) with lot number #12021401 and #05011702 was calibrated using the INTRABEAM X‐ray source (Carl Zeiss Surgical, Oberkochen, Germany).

### 2.1 EBT3 film preparation and calibration

The EBT3 film is a transparent film consisting of a single active layer with a thickness of 30 µm that is coated to 125 and 125 µm thickness of double matte polyester layer, respectively.^23^ The EBT3 films were cut into square pieces of 20 × 20 mm^2^.

The films were irradiated using the INTRABEAM 50 kV photon beam in a water phantom for calibration. The EBT3 film was set at a source‐to‐detector distance (SDD) of 10 mm in the water phantom on the ionization chamber holder by putting the EBT3 film on the top surface of the holder. The films were irradiated to known doses of 1–25 Gy at selected intervals. Since two boxes of EBT3 films were used for this work, separate calibration curves were established for each box of films.

The films were scanned 24 hours after irradiation to allow for post‐irradiation color changes, using an Epson 10000 XL flatbed scanner (Epson America Inc, Long Beach, CA). The films were scanned in transmission mode at 75 dots per inch (dpi) and 48 bits RGB. The images of the scanned film were saved in TIFF format to avoid compression and loss of data. The films were scanned in the central region of the scanner to reduce the effect of scanner non‐uniformity.^24^ All the images were analyzed using the ImageJ 1.47 software (National Institution of Health, USA). The green channel was used in this work because the red channel has a limited dose range of 8 Gy, while the green channel has been reported to have a usable range up to 40 Gy.^1^, ^16^

Three pieces of films were used for measurement settings. The mean and 1 standard deviation (1 SD) of the mean pixel value or dose values were presented.

### 2.2 Patient recruitment

A total of 21 patients with early breast cancer treated with BCS with IORT alone or IORT boost plus EBRT as per TARGIT‐A and TARGIT‐B protocols from June 2016 to April 2018 were recruited in this prospective study. A prescribed dose of 20 Gy was delivered using the INTRABEAM system (Carl Zeiss Surgical, Oberkochen, Germany) to the applicator surface in a single fraction.

The study was approved by the local institutional review board (MECID.NO: 2015121958) with written consent obtained from patients. Patient demographics, clinical characteristics (tumor grade, size, hormonal receptors, lymphovascular invasion, nodal status, stage (TNM), and ER/PR/HER2 status), treatment data (chemotherapy, endocrine therapy, and targeted therapy), and local complications (such as skin toxicity, surgical site infection, seroma, and other types of complications) were recorded. The mid‐term skin toxicity was evaluated at two time‐points; at 3 and 6 months post‐IORT, respectively. The skin toxicity was evaluated according to the Radiation Therapy Oncology Group (RTOG) toxicity grading.^25^

Details of IORT and EBRT were also reported. The spherical applicator's size during the IORT procedure was determined by the size of the cavity created due to lumpectomy. Skin toxicity was minimized by ensuring that at least 10 mm of breast tissue was present between the applicator's surface and the skin, as suggested by Keshtgar *et al*. (2014).^26^

### 2.3 In vivo dosimetry during IORT to measure skin dose

**FIGURE 1:**
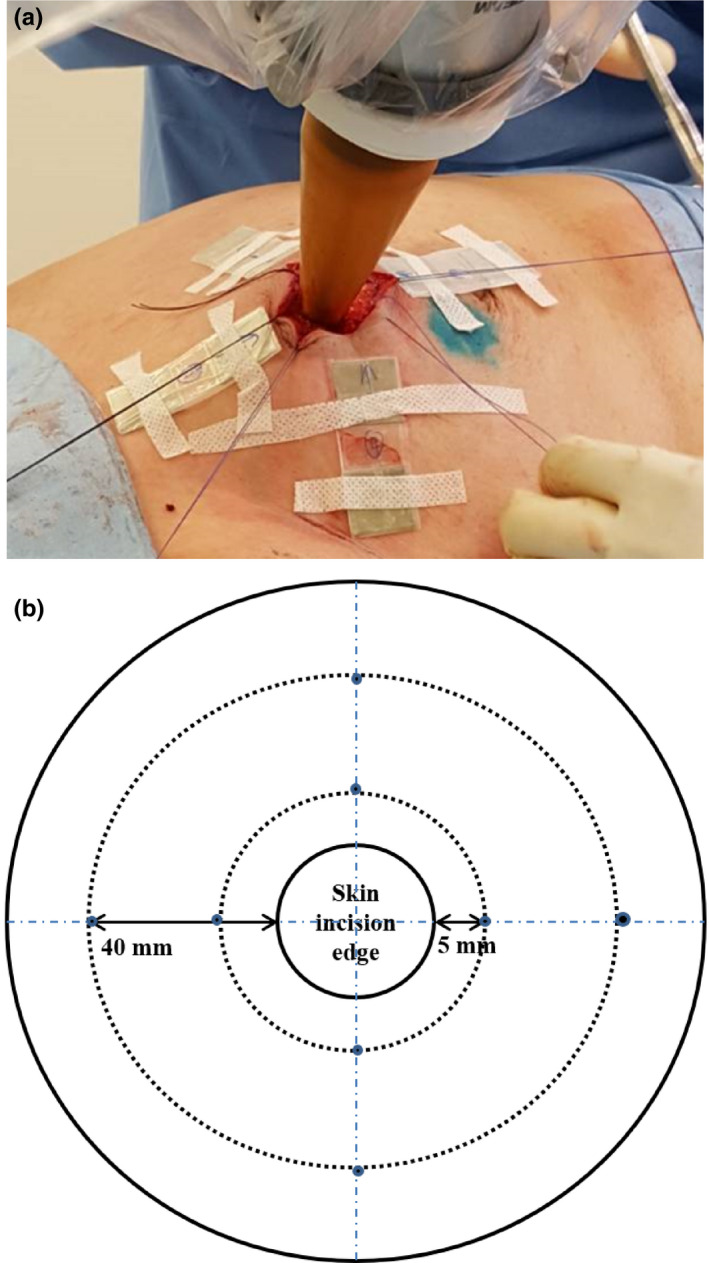
(A) Eight pieces of EBT3 films on the patient's skin during the IORT procedure with the “pulling‐string” suture applied and (B) schematic diagram showing the film's placement.

Skin doses of the patients undergoing IORT treatment after lumpectomy during BCS were measured using the EBT3 film. Eight pieces of the EBT3 films, cut to 20 × 20 mm^2^, were placed on the skin surface. Four pieces of film were positioned with the inner edge flushed 5 mm from the skin edge enclosing the applicator stem in the superior, inferior, medial, and lateral directions. The outer four pieces of film were placed 40 mm away from the skin edge enclosing the applicator stem in the superior, inferior, medial, and lateral directions (Figure 1). Once all EBT3 films had been taped in place, “pull‐string” sutures were used to gently retract the skin away from the applicator stem in an attempt to reduce skin toxicity.^27^

For patients who underwent EBRT, the maximum skin dose was estimated from the radiotherapy treatment planning system.^28^, ^29^ A 2‐mm skin contour was made on the irradiated breast using Eclipse radiotherapy treatment planning system (TPS) version 13 (Varian Medical Systems, Inc., Palo Alto, CA, USA). The maximum absorbed dose at 0.03 mm^2^ skin volume was obtained from the dose‐volume histogram (DVH).

### 2.4 Statistical analysis

Statistical analysis was performed using IBM SPSS, version 22 (IBM Corp, Armonk, NY, USA). The numerical data were tested for normality using the Shapiro–Wilk test. Data descriptive summary was reported using mean ±1 SD for normally distributed data, while median and interquartile range (IQR) were reported for those that were not normally distributed.

*t*‐test was used to assess the mean difference of the skin doses concerning the development of seroma. One‐way analysis of variance (ANOVA) was used to assess skin doses’ mean difference with skin toxicities.

Correlation between treatment time, applicator size, tumor size, skin doses, and skin toxicities were assessed using Spearman's correlation. The association between nominal clinical parameters and skin toxicities was assessed using the Chi‐square test of independence.

## 3 RESULTS

### 3.1 EBT3 film calibration curve

**FIGURE 2:**
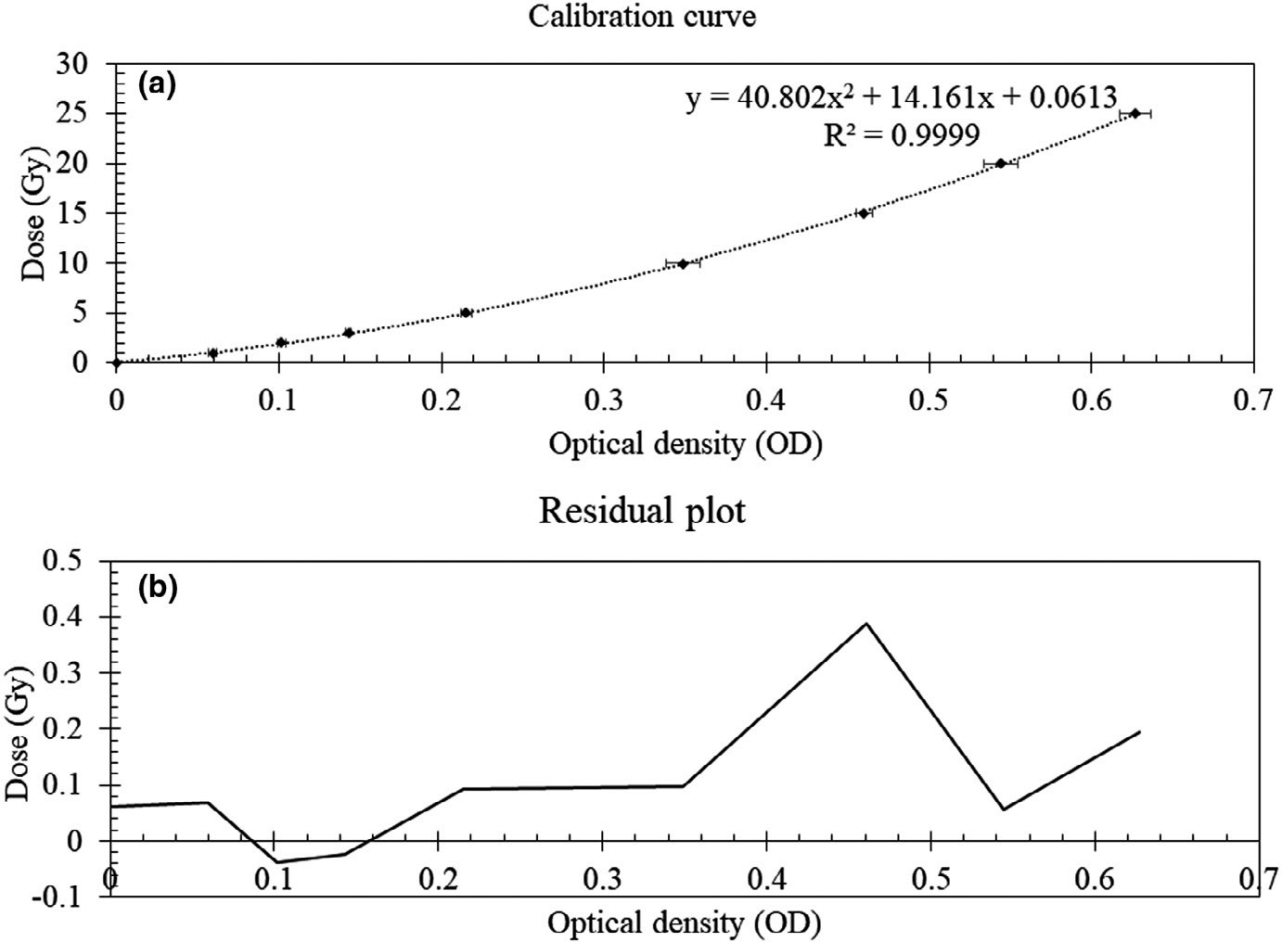
(A) Calibration curve of the EBT3 film as a function of dose (Gy) versus optical density (OD) and (B) the residual plot.

A calibration curve of the EBT3 film relating the dose of up to 25 Gy for 50 kV photon beam is shown in Figure 2. The calibration curve was established using the green channel. A second‐degree polynomial fit was fitted to the calibration curve. The error bar represents the mean and 1 SD of the mean of three sets of measurement. The average coefficient of variation is 2.1%.

### 3.2 Patients and treatment characteristics

Table 1 summarizes the patients’ clinicopathological and treatment characteristics. The average age was 58 ± 9 (range 42–71 years). All patients were diagnosed with invasive ductal carcinoma and had hormone receptor‐positive disease. The average tumor size was 16.8 ± 10.0 mm. The mean follow‐up time was 37 ± 8 months, and no local recurrences were reported. Among the eight patients with lymphovascular invasion, six had no lymph node involvement. The other two patients each had between one and three lymph nodes involved.

**TABLE 1:** Clinicopathological and treatment characteristics.

Description	N (%) or Mean ±1SD	
Number of subjects, N	21 (100)
Age (year)	58 ± 9	
Tumor size (mm)	16.8 ±10.0
Grade		
1	4 (19.0)	
2	16 (76.2)	
3	1 (4.8)	
Tumor staging	
Stage 1	13(62)	
Stage 2	8 (38)	
Lymphovascular invasion	
No	13 (62)	
Yes	8 (38)	
Estrogen receptor	
ER‐	3 (14)	
ER+	18 (86)	
Progesterone receptor	
PR‐	4 (19)	
PR+	17 (81)	
Human epidermal growth factor receptor 2 (HER2 immunohistochemistry)		
HER2 –	14 (67)	
HER2 2+	3 (14)	
HER2 3+	4 (19)	
Neoadjuvant chemotherapy (NAC)	
No	16 (76)	
Yes	5 (24)	
Tamoxifen	
No	2 (10)	
Yes	19 (90)	

Table 2 summarizes the radiotherapy details of IORT and EBRT. Sixty‐four percent (9 / 14) of patients who had planned to undergo IORT alone did not need EBRT, while five were subjected to EBRT as an adaptive‐risk approach. All patients who underwent EBRT had 40 Gy delivered in 15 fractions to the whole breast. The median interval time from IORT to the delivery of EBRT was 8.4 weeks (ranged 5.8–30.6 weeks). The applicator size ranged from 35 to 50 mm, with the average treatment time of 32.3 ± 10.2 minutes.

**TABLE 2:** Details of patients undergoing IORT, IORT boost, and EBRT.

Description	N (%) or Mean ±1SD	
IORT indications		
IORT alone	14 (67)	
IORT boost	7 (33)	
External beam radiotherapy (EBRT)	
No	9 (43)	
Yes	12 (57)	
IORT applicator diameter (mm)		
35	2 (10)	
40	7 (33)	
45	5 (24)	
50	7 (33)	

Table 3 shows the treatment‐related complications. At 3 months post‐IORT, 86% of patients showed no evidence of skin toxicity. However, the number of patients showing skin toxicity increased from 15% to 38% at 6 months post‐IORT. The toxicity rate was 11% for IORT alone compared with IORT boost plus EBRT (17%) at 3 months post‐IORT. At 6 months post‐IORT, the toxicity rate increased to 22% for IORT alone, and 50% for IORT boost plus EBRT. None of the other clinical parameters were associated with skin toxicity.

**TABLE 3:** Treatment‐related complications.

Description	N (%) or Mean ±1SD	
Surgical site infection (SSI)/seroma	
No	15 (71)	
Yes	6 (29)	
Skin toxicity (RTOG grades)		
@ 3 months		
0	18 (86)	
1	2 (10)	
2	1 (5)	
@ 6 months		
0	13 (62)	
1	7 (33)	
2	1 (5)	

### 3.3 Skin dose

The average skin doses received during IORT, measured at a distance of 5 and 40 mm away from the applicator, were normally distributed. The treatment time and applicator and tumor sizes were not normally distributed.

Table 4 shows the average skin dose received by patients who underwent IORT alone and IORT boost plus EBRT. The average skin dose at 5 and 40 mm away from the applicator was 3.07 ± 0.82 Gy and 0.99 ± 0.28 Gy, respectively. Patients treated with IORT boost plus EBRT received an additional skin dose of 41.07 ± 1.57 Gy.

**TABLE 4:** Skin dose due to IORT and EBRT, presented as mean ±1SD.

Type of radiotherapy	Skin dose (Gy)	
Intraoperative radiotherapy	Without EBRT (n = 9)	With EBRT (n = 12)
@ 5 mm distant from skin edge	3.04 ± 1.01	3.10 ± 0.69
@ 40 mm distant from skin edge	1.04 ± 0.28	0.96 ± 0.28
External beam radiotherapy	Not applicable	41.07 ± 1.57

Skin dose from IORT was neither significantly higher in patients with seroma, nor was it different between the type of treatment in cases of skin toxicity. Although patients treated with IORT boost plus EBRT received a much higher dose, the incidences of seroma and skin toxicity were not significantly different from those treated with IORT alone. One patient received a skin dose of 5.12 ± 1.63 Gy during IORT treatment but did not suffer from any toxicity.

The skin toxicity at 6 months was significantly correlated with older patients (r = 0.5, *p *= 0.021). IORT treatment time was positively correlated with applicator (r = 0.956, *p *< 0.001) and tumor (r = 0.492, *p *= 0.023) sizes. The average skin dose at 40 mm away from the applicator was moderately correlated with treatment time (r = 0.53, *p *= 0.013) and applicator size (r = 0.501, *p *= 0.021). However, no correlation was found between the skin toxicity with treatment time, applicator size, tumor size, and average skin dose at 5 and 40 mm away from the applicator.

## 4 DISCUSSION

### 4.1 EBT3 film dosimetry

Gafchromic films in kV energy beams often give rise to the question of energy dependence, both with respect to the megavoltage beams (e.g., 6 MV–15 MV photon beam) and the beam hardening effect.^15^, ^19^, ^20^, ^30^, ^31^ Monte Carlo simulations by Moradi et al. had shown that the 50 kV beam hardens with the use of spherical applicators and distance from the tip of the XRS source.^32^

The energy dependence of Gafchromic films also varied with different types, generally categorized by the radiology films (XR‐series) and the radiotherapy films (EBT‐series).^15^, ^19^, ^20^, ^30^, ^31^ Most studies compared the energy dependence of the film at kV beams to megavoltage beams, reporting a large energy dependence of up to 11%–20%.^19^, ^20^ In radiotherapy, calibration was typically carried out using megavoltage beams, whereby Compton scattering dominates the photon interaction. However, the detector's response is often different at the kV energy range due to the dominant photoelectric absorption effect. To address the energy‐dependent problem, it is crucial to calibrate the film in the clinical beams used. This approach has been applied when using radiation detectors known to be energy dependent when used under kV photon beams.^22^, ^33^ The energy dependence of the radiation detectors calibrated under the clinical beams would be much lesser within the energy range used. Brown et al.^34^ reported a relative sensitivity of 3% for EBT3 film used under 25–35 keV monochromatic beams. Villarreal‐Barajas et al.^20^ reported response variation of ~3% for 32–38 keV effective energies (equivalent to 70–100 kVp) polychromatic X‐ray beams, within the same film batch.

In our study, we calibrated the EBT3 films at 10 mm from the tip of the source. Based on Moradi et al.,^32^ the effective beam energy is 28.47 keV at this depth. Upon application of the spherical applicators, the effective beam energies varied from 27.8 to 29 keV for the applicator size of 15 mm–50 mm. Moradi et al. also demonstrated good agreement of the EBT3 film depth dose measurement with Monte Carlo calculation. This indicates that the EBT3 film is not sensitive to the slight beam hardening effect due to depth or distance variation to the source.

These data show that the calibration curve derived using the 50 kV beam in water is suitable for in vivo dosimetry.

In this work, the calibration curve was established using the green channel instead of the red channel that is conventionally used in most radiochromic film work. This is because the green channel produced a curve with a shallower slope, allowing it to be used up to a much higher dose level (>8 Gy).^35^ Villarreal‐Barajas et al. ^20^ showed that using the green channel resulted in 2.5% uncertainty. They also reported that the batch‐to‐batch variability for the green channel was better than the red channel at kV beams.^20^ However, in this work, the calibration curve was established for each box of films to reduce the batch‐to‐batch variation.

The uncertainty budget of using EBT3 film for in vivo dosimetry of breast IORT was determined by taking into account the EBT3 film reproducibility, beam hardening effect, and the uncertainty related to the use of the green channel (Table 5). The total uncertainty of 4.4% was obtained by adding the selected uncertainties in quadrature. The expanded uncertainty (k = 2) is 8.8%.

**TABLE 5:** Summary of uncertainties for dose measurement and the total dose uncertainty.

Physical Aspects	Type of uncertainty	Uncertainty (%)
EBT3 film reproducibility	Type A	2.1 (This work)
Energy dependence / Beam hardening effect	Type A	3.0 (Brown et al.^34^)
Green channel	Type A	2.5 (Villarreal‐Barajas et al.^20^)
$U_{\mathrm{total}}$(k=1)		4.4
(k=2)		8.8

### 4.2 Radiation‐induced skin toxicity due to IORT

Radiation‐induced skin toxicity is a well‐known complication arising from radiation therapy. Our study found that the average maximum skin dose in IORT was 3.05 Gy, while the average maximum daily skin dose received from EBRT was 2.94 Gy. Acute radiation doses might cause skin erythema at 2 Gy and permanent epilation at 7 Gy.^36^

During IORT, a large dose of 20 Gy was delivered to the tumor bed. Thus the skin has been exposed to a higher radiation dose. For patients that underwent EBRT, Skin tissue is more tolerant toward radiation doses delivered in a fractionated manner. For example, when the dose was delivered at 40 Gy in 15 fractions, this would allow partial healing to occur.^37^

However, the cumulative dose from multiple radiation treatment at the same site might lead to an increased risk of tissue damage. The EBRT dose measurement was comparable to those reported by Jong *et al*. (2016).^28^ This dose level was quite tolerable for skin tissue, but the tissue at the exposed site would require time to repair and recover from the daily radiation injury. This study was limited by the small sample size and the lack of in vivo skin dose measurements in EBRT. The EBRT dose was obtainable only from the treatment planning system, which might be inaccurate for superficial skin dose estimation due to the limitation of the pencil beam convolution dose calculation algorithm.^28^, ^29^

The radiation's cumulative damage on skin tissue, coupled with the individual clinical characteristics, may cause some patients to develop grade 1 and 2 skin toxicity at a later stage of treatment. No correlation was observed between skin toxicity and the dose received. The median interval time from IORT to EBRT of 8.4 weeks might have allowed some tissue healing to occur. Given that the interval time from IORT to the delivery of EBRT ranged from 5.8 to 30.6 weeks, there was a possibility that one of the skin toxicity was assessed during the course of the EBRT delivery. In which case, the skin toxicity might have occurred due to the acute effect of EBRT. Careful screening of EBRT treatment dates revealed that 43% (three out of seven cases) fell within this time frame. Thus, skin toxicity as a result of acute effects in EBRT could not be completely ruled out.

The only patient‐related characteristic that was found to be significantly correlated with late skin toxicity was age. Older patients in poor health might have a lower ability to heal, resulting in more severe skin reactions at the 6‐month assessment.

**FIGURE 3:**
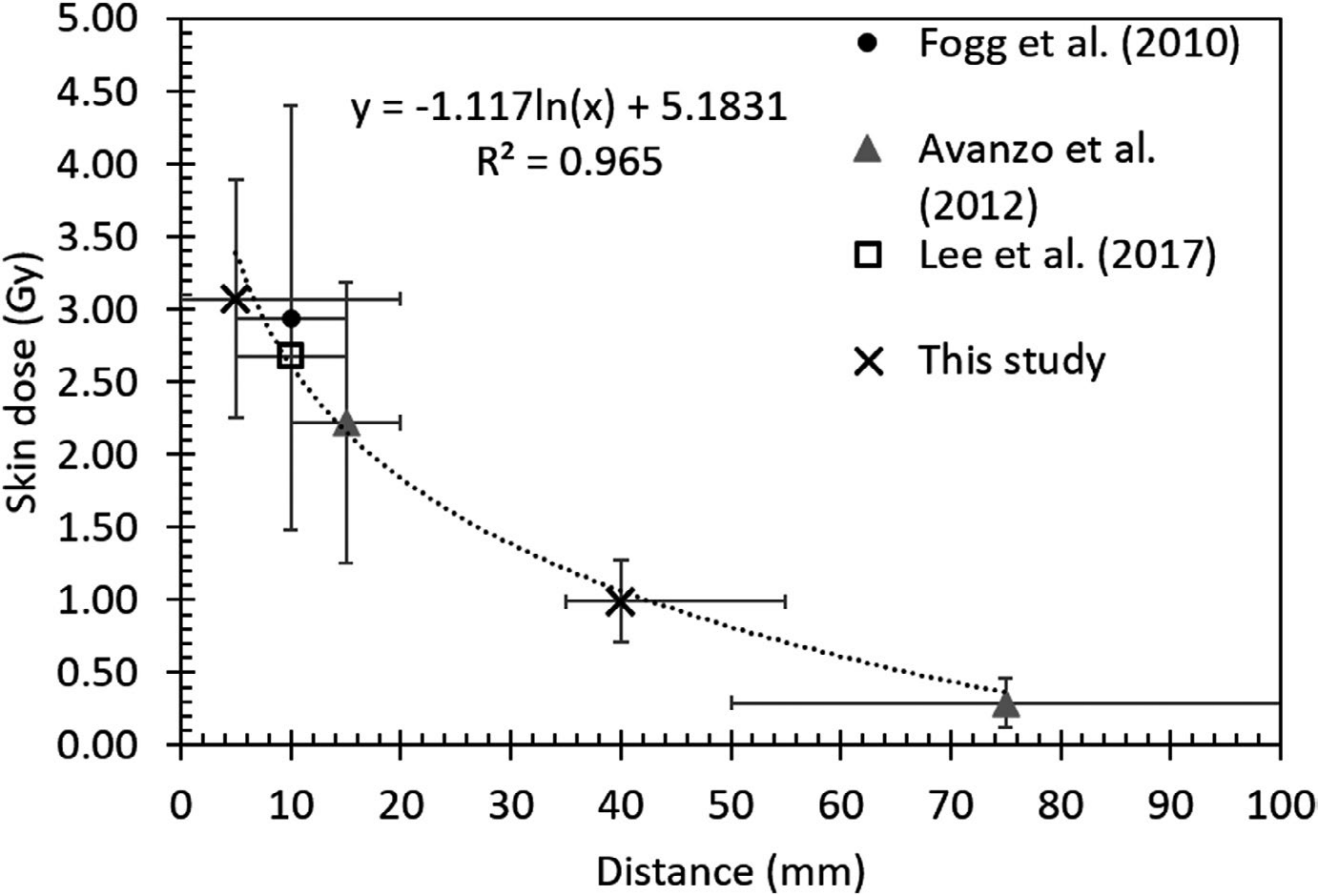
Comparison of the measured skin doses from INTRABEAM IORT with similar studies. The measurement points are placed in the middle of the measured region, while the horizontal error bars represent the length of measurement regions. The vertical error bars represent ±1 SD of the mean skin dose. The dotted line is the logarithmic fit of data points.

The skin dose measurement from our study was comparable to previous studies.^12^, ^13^, ^14^ Figure 3 consolidated the previous studies’ and the current findings, providing an unprecedented insight into the skin dose fall‐off due to IORT.^12^, ^13^, ^14^ An empirical logarithmic model to predict skin dose was derived as a function of distance from the source/applicator, as shown in Equation 1.

(1) $$\text{Skin}\;\text{dose}\left(\text{in}\;Gy\right)=‐1.117\ln\left(\text{distance}\;\text{from}\;\text{radiation}\;\text{source}\;\text{in}\;\text{mm}\right)+5.1831$$

The skin dose showed a logarithmic dose fall‐off with distance from the applicator. The more considerable uncertainties observed at shorter distances to the applicator may be caused by positioning uncertainties. At further distances from the applicator, the dose uncertainties are lower at the flatter dose fall‐off region.

## 5 CONCLUSION

The EBT3 film can be used as a dosimeter for in vivo skin dosimetry in IORT to provide direct patient‐specific skin dose information. We found that patients who developed skin toxicity did not necessarily receive significantly higher skin dose (in IORT boost plus EBRT). Patients who underwent IORT boost plus EBRT also did not show a significantly higher incidence of skin toxicity. Given the extended time interval between IORT and EBRT, the expression of a more severe form of skin reaction may be reduced. The later onset of skin toxicities might be associated with old age. We had developed a model to predict skin dose in IORT. It was reassuring to see that IORT alone and IORT boost plus EBRT were relatively safe in terms of skin toxicity. This would provide clinicians with more treatment options for patients who were considered for IORT after BCS.

## CONFLICT OF INTEREST

The authors declare they have no conflict of interest.

## AUTHORS’ CONTRIBUTIONS

Jeannie Wong Hsiu Ding analyzed the data, perform statistical analysis, and drafted the manuscript. Zainor Zaili performed the dose measurement and film analysis. Ung Ngie Ming, Rozita Abdul Malik, Anita Zarina Bustam and, Marniza Saad involved in the data collection of the skin toxicity and radiotherapy treatment planning. Suniza Jamaris and Joanne Aisha Mosiun collected the patient data. Nur Aishah Mohd Taib conceptualized the study editing and authorizing final manuscript. Mee Hoong See and Ung Ngie Ming conceptualized the study, supervised the study, collecting the data, final re‐edit the manuscript, re‐editing, and authorizing the final manuscript. All authors finalized the manuscript.

## ETHICS APPROVAL

All procedures performed in studies involving human participants were in accordance with the ethical standards approved by the local institutional review board (MECID.NO: 2015121958) of Medical Ethics Committee, University Malaya Medical Center.

## Data Availability

Data available on request from the authors.
